# Tree-Based Classification of COVID-19 Using NanoString Whole-Blood Immune-Response Profiles: Comparison of Full-Dataset and LOOCV-Embedded Feature Selection

**DOI:** 10.3390/v18091009

**Published:** 2026-09-13

**Authors:** Zeynep Burcin Yilmaz, Zeynep Kucukakcali, Sami Akbulut

**Affiliations:** 1 Department of Infectious Diseases and Clinical Microbiology, Inonu University Faculty of Medicine, Malatya 44280, Türkiye; burcin.yilmaz@inonu.edu.tr; 2 Department of Biostatistics and Medical Informatics, Inonu University Faculty of Medicine, Malatya 44280, Türkiye; zeynep.tunc@inonu.edu.tr; 3 Department of Surgery and Liver Transplant Institute, Inonu University Faculty of Medicine, Malatya 44280, Türkiye; 4 Department of Public Health, Inonu University Faculty of Medicine, Malatya 44280, Türkiye

**Keywords:** COVID-19, whole-blood transcriptomics, machine learning, cross-validation-embedded feature selection, leave-one-out cross-validation

## Abstract

Background: Whole-blood transcriptomic profiling can capture systemic immune-response alterations associated with COVID-19 and may support host-response-based classification. However, evidence regarding the discriminatory value of targeted immune-gene panels remains limited, and in small, high-dimensional datasets, the timing of feature selection may substantially affect model performance and interpretation. Aim: This study aimed to evaluate whether NanoString Human Immunology Panel profiles could distinguish COVID-19 from healthy-control measurements and to compare full-dataset feature selection (FDFS) with leave-one-out cross-validation (LOOCV)-embedded feature selection (LEFS). Methods: Publicly available E-MTAB-8871 data comprising 579 genes and 32 whole-blood transcriptomic profiles were analyzed. The dataset included 22 longitudinal COVID-19 measurements obtained from three participants and 10 measurements obtained from 10 healthy controls. Elastic Net regularization was used for feature selection. Random Forest, XGBoost, and LightGBM classifiers were evaluated using sample-level LOOCV. Model performance was assessed using threshold-dependent, discrimination, and probability-based metrics. A separate exploratory LightGBM model was analyzed using SHapley Additive exPlanations (SHAP) to characterize feature contributions. Results: FDFS identified a fixed 40-gene set, whereas LEFS selected a mean of 42 genes per fold (range: 40–47). LightGBM correctly classified all 32 measurement-level profiles (derived from 13 unique participants: 10 healthy controls and three longitudinally sampled COVID-19 participants) in both frameworks, achieving area under the receiver operating characteristic curve (ROC-AUC) and area under the precision–recall curve (PR-AUC) values of 1.000 and Brier scores of 0.005 and 0.006 in the FDFS and LEFS frameworks, respectively. Random Forest achieved accuracies of 0.969 and 1.000, whereas XGBoost achieved an accuracy of 0.969 in both frameworks. SHAP analyses consistently identified *AICDA* as the dominant contributor to model predictions, followed by *ARHGDIB*. Conclusions: This exploratory analysis showed that targeted NanoString immune-response profiles contained a compact transcriptomic signal capable of distinguishing COVID-19 from healthy-control measurements within the analyzed dataset. These findings provide proof-of-concept evidence of internal measurement-level discrimination. However, because the COVID-19 profiles consisted of repeated measurements from only three participants, sample-level LOOCV did not constitute independent participant-level validation. External validation in larger cohorts comprising independently sampled participants is required. Given that the COVID-19 arm comprised only three independent participants, these biological findings should be regarded as hypothesis-generating and require validation in substantially larger independent cohorts.

## 1. Introduction

Coronavirus disease 2019 (COVID-19) is caused by severe acute respiratory syndrome coronavirus 2 (SARS-CoV-2), an enveloped, positive-sense single-stranded RNA betacoronavirus that emerged in late 2019 [1,2,3,4]. Its rapid international spread created a global health emergency, disrupted healthcare delivery and utilization, and generated substantial direct and indirect health burdens [2,3,4,5]. World Health Organization (WHO) excess-mortality analyses estimated 14.83 million excess deaths during 2020–2021 alone, compared with 5.42 million reported COVID-19 deaths for the same period, indicating that the total direct and indirect mortality burden of the pandemic substantially exceeded officially reported deaths [4]. By 2024, more than 770 million confirmed infections and nearly 7 million deaths had been reported worldwide [2]. Although vaccination, population immunity, viral evolution, and advances in clinical management have altered the epidemiology of COVID-19, the disease remains clinically relevant because acute outcomes and post-acute consequences continue to vary substantially across patient groups [2,6,7,8].

COVID-19 is characterized by marked clinical heterogeneity. Individuals may remain asymptomatic or experience mild respiratory illness, whereas others develop pneumonia, acute hypoxemic respiratory failure, acute respiratory distress syndrome, thrombotic or coagulation complications, multiorgan dysfunction, or death [3,6]. Demographic characteristics and pre-existing diseases contribute to this variability but do not fully explain why apparently similar patients follow different trajectories [6,9]. Evidence therefore indicates that disease expression reflects not only viral replication but also the magnitude, composition, and timing of the host response [10,11,12]. Defining infection-associated molecular patterns is fundamental to understanding the host response to SARS-CoV-2, identifying disease-associated biological pathways, and developing complementary host-response-based tools for infection-status classification [13,14,15].

SARS-CoV-2 infection can induce dysregulated innate and adaptive immune responses. Transcriptomic and immunophenotypic studies have described impaired or delayed type I and III interferon responses in some patients together with increased chemokine and inflammatory cytokine signaling. Other studies have further shown altered myeloid-cell activity, neutrophil-associated inflammatory programs, lymphocyte depletion, and changes in B- and T-cell phenotypes, highlighting the complexity and dysregulation of the host response [10,11,12,16,17,18]. These responses are not static. Longitudinal investigations have shown that inflammatory, antiviral, and lymphocyte-associated transcriptional programs change across the disease course, may peak at different phases of respiratory deterioration and recovery, and can remain perturbed beyond acute infection [12,16,19]. This temporal and interindividual heterogeneity limits the information obtainable from isolated biomarkers and supports the evaluation of multigene immune-response patterns.

Peripheral blood transcriptomics provides a minimally invasive approach for investigating systemic host responses to infection. Gene-expression profiles from circulating leukocytes can capture coordinated immune-response programs, including interferon-related, inflammatory, adaptive immune, and convalescence-associated signals [13,17,19]. Compared with genome-wide RNA sequencing (RNA-seq), targeted digital expression platforms offer a more focused and potentially translationally relevant strategy for immune profiling [20]. The NanoString nCounter system directly quantifies predefined transcripts through color-coded probe pairs without enzymatic amplification and can measure hundreds of immune-related genes from small amounts of RNA [20,21]. Targeted panels therefore bridge the analytical space between discovery-oriented transcriptomics and low-plex quantitative polymerase chain reaction (qPCR) assays [19,20,21].

However, the ability to quantify hundreds of immune-related transcripts simultaneously also generates a high-dimensional analytical problem, particularly when the number of biological samples is limited. Gene-expression datasets commonly contain hundreds or thousands of correlated predictors but few biological samples, producing a high-dimensional *p* ≫ n structure that increases instability and susceptibility to overfitting. Feature selection and regularization are therefore important for reducing redundancy and constructing parsimonious signatures. Machine-learning (ML) methods, including Random Forests and gradient-boosted decision trees, are attractive because they can accommodate nonlinear relationships, interactions, and correlated predictors. Previous transcriptomic ML studies in COVID-19 have focused mainly on disease severity, prognostic stratification, complementary host-response diagnosis, and differential blood biomarker discovery using heterogeneous RNA-seq or microarray datasets [9,13,14,18,22,23]. Among the studies reviewed here, most investigated genome-wide RNA-seq or microarray data, disease severity or prognosis, biomarker discovery, or targeted-panel development; none directly compared full-dataset and cross-validation-embedded feature selection in this specific small-sample NanoString whole-blood setting [9,13,14,18,20,22,23].

A central methodological concern is that feature selection constitutes part of model training [24,25,26]. Selecting genes from the complete dataset before cross-validation allows information from held-out observations to influence the predictor set, creating data leakage and potentially optimistic performance estimates [24,25,26,27]. In small-sample omics studies, data-dependent operations—including scaling, feature selection, and tuning—should therefore be restricted to the training partition and repeated within each resampling iteration to reduce preprocessing leakage and optimistic bias in performance estimation [24,25,28]. Globally selected gene sets may still support exploratory biological interpretation, provided that their performance is not presented as fully independent validation [24,25]. Comparing global and cross-validation-embedded feature-selection frameworks can therefore clarify the distinction between signature discovery and internal classification performance evaluation [24,25,26].

Beyond discrimination, model interpretation can help determine whether predictions depend predominantly on a small subset of influential features or are distributed across a broader set of variables [29]. High classification accuracy alone does not establish biological validity [30] or generalizability to independent data [25]. SHapley Additive exPlanations (SHAP) assign feature-level contributions to individual predictions and can aggregate local explanations to characterize global behavior in tree-based models [29]. The sign of a SHAP value indicates whether a feature pushes the model output toward or away from the predicted class; however, SHAP values should not be interpreted as evidence of differential-expression direction, biological causality, or mechanism [29,30]. Therefore, in this exploratory study, we aimed to determine whether NanoString-based immune-response transcriptomic profiles could distinguish COVID-19 measurements from healthy-control measurements, compare full-dataset feature selection with leave-one-out cross-validation-embedded feature selection across tree-based ensemble classifiers, and characterize gene-level contributions to model predictions.

## 2. Materials and Methods

### 2.1. Dataset Characteristics

#### 2.1.1. Data Source and Acquisition

Gene-expression profiles from individuals with COVID-19 and healthy controls were analyzed using the publicly available BioStudies/ArrayExpress dataset E-MTAB-8871 [31]. The study is registered as a transcription profiling by array experiment in Homo sapiens using the NanoString Human Immunology Panel. According to the repository metadata, the study combines a case–control design with a time-series design, and its main experimental factors are defined as disease, individual, and time. This structure is directly relevant to the present analysis because the COVID-19 group was sampled longitudinally rather than at a single time point.

#### 2.1.2. Repository Content and Analytical Files

The repository provides both raw and processed data. The raw archive contains 33 NanoString Reporter Code Count (RCC) files, whereas the processed archive contains the normalized expression matrix nCOV2_Norm_log2_counts.txt together with the Sample and Data Relationship Format (SDRF) file, E-MTAB-8871.sdrf.txt, and the Investigation Description Format (IDF) file, E-MTAB-8871.idf.txt. The SDRF file supplies sample-level annotation, including disease status, subject identifier, sex, age, and sampling time, while the IDF file summarizes the overall study design, protocols, and processing steps. For the present study, the main analytical dataset was the processed expression matrix, and the SDRF/IDF files were used to verify sample identity, disease grouping, and experimental procedures.

#### 2.1.3. Experimental Procedures and Preprocessing Context

According to the repository protocol, 3 mL of whole blood was collected in Tempus Blood RNA tubes (Applied Bio-systems). RNA was extracted using the Tempus Spin RNA Isolation Kit (Invitrogen) in accordance with the manufacturer’s instructions. No nucleic acid labeling step was applied. For hybridization, 50 ng of total RNA was incubated with reporter and capture probe sets at 65 °C for 24 h. Hybridized samples were then loaded onto the nCounter cartridge, and post-hybridization processing and scanning were performed using the nCounter Sprint Profiler. Raw RCC files were analyzed with nSolver Analysis Software version 4.0 (NanoString Inc.) according to the manufacturer’s protocol. The repository further indicates that negative control probes were used for background thresholding, positive control probes were used to normalize variation in hybridization efficiency, and internal reference genes were used to normalize differences in sample input [16].

#### 2.1.4. Processed Expression Matrix

The processed file used in this study, nCOV2_Norm_log2_counts.txt, contains normalized log2-transformed expression values. Structurally, the file includes one identifier column and 32 sample columns. The first two rows store sample-level descriptors, namely Time and Disease, and the remaining 579 rows correspond to genes. Thus, the analytical expression matrix used in the present study consisted of 579 gene-level measurements across 32 analyzed samples. No missing observations were identified in the processed matrix.

#### 2.1.5. Sample Composition and Longitudinal Structure

The analytical dataset comprised 32 samples, divided into 22 COVID-19 samples and 10 healthy control samples. In the classification framework, COVID-19 samples were coded as the positive class, whereas healthy controls were coded as the negative class. The 10 control samples each represented a separate healthy control individual. In contrast, the 22 COVID-19 samples were obtained longitudinally from three COVID-19 cases sampled at multiple time points during the disease course. Based on the processed matrix and SDRF metadata, the COVID-19 sampling schedule can be summarized as follows: Case 1 was sampled on days 4, 5, 6, 7, 8, 9, 11, 12, and 18; Case 2 was sampled on days 6, 7, 8, 9, 10, 11, 12, 13, and 19; and Case 3 was sampled on days 9, 10, 11, and 12. Accordingly, the dataset contains 32 analyzed samples, but these do not represent 32 distinct participants. At the participant level, the dataset corresponds to 13 unique individuals, consisting of 10 healthy controls and 3 COVID-19 cases. Therefore, the analytical matrix should not be interpreted as 32 statistically independent observations, because the COVID-19 arm is composed of repeated measurements from only three participants. Thus, the nominal analytical sample size was *n* = 32 measurement-level profiles, whereas the effective biological sample comprised 13 independent participants. Importantly, for estimation of the COVID-19-associated classification signal, the effective number of independent COVID-19 biological units was limited to only three participants.

#### 2.1.6. Clarification of the Reported Sample Count

The BioStudies/ArrayExpress study page reports a sample count of 33, which initially appears inconsistent with the processed matrix. This discrepancy is explained by the SDRF file, where Case 1 at day 4 is represented by two entries, Case1_d4_rep1 and Case1_d4_rep2, indicating a technical replicate structure in the raw metadata. By contrast, the processed file used for analysis contains 32 final sample columns, indicating that the analytical matrix reflects a consolidated processed representation rather than 33 separate analyzed profiles.

#### 2.1.7. Analytical Implications for Model Development

The final dataset has a clear high-dimensional structure, with 579 gene-level predictors and 32 analyzed expression profiles, corresponding to a typical *p* ≫ *n* setting. However, the profile count overstates the effective number of independent observations, because 22 COVID-19 profiles arise from repeated measurements in only three participants. This structure increases the risk of model instability and overfitting if all predictors are used without restriction. For this reason, downstream analyses incorporated explicit feature selection and complexity-controlled modeling strategies, and cross-validation results were interpreted with consideration of the repeated-measures structure of the dataset. Overall, E-MTAB-8871 provides a compact but information-rich whole-blood NanoString dataset that combines longitudinal sampling in COVID-19 cases with healthy control comparators. The availability of raw RCC files, processed normalized counts, and structured metadata supports transparent reanalysis and exploratory transcriptomic investigation.

### 2.2. ML Framework

**Figure 1:**
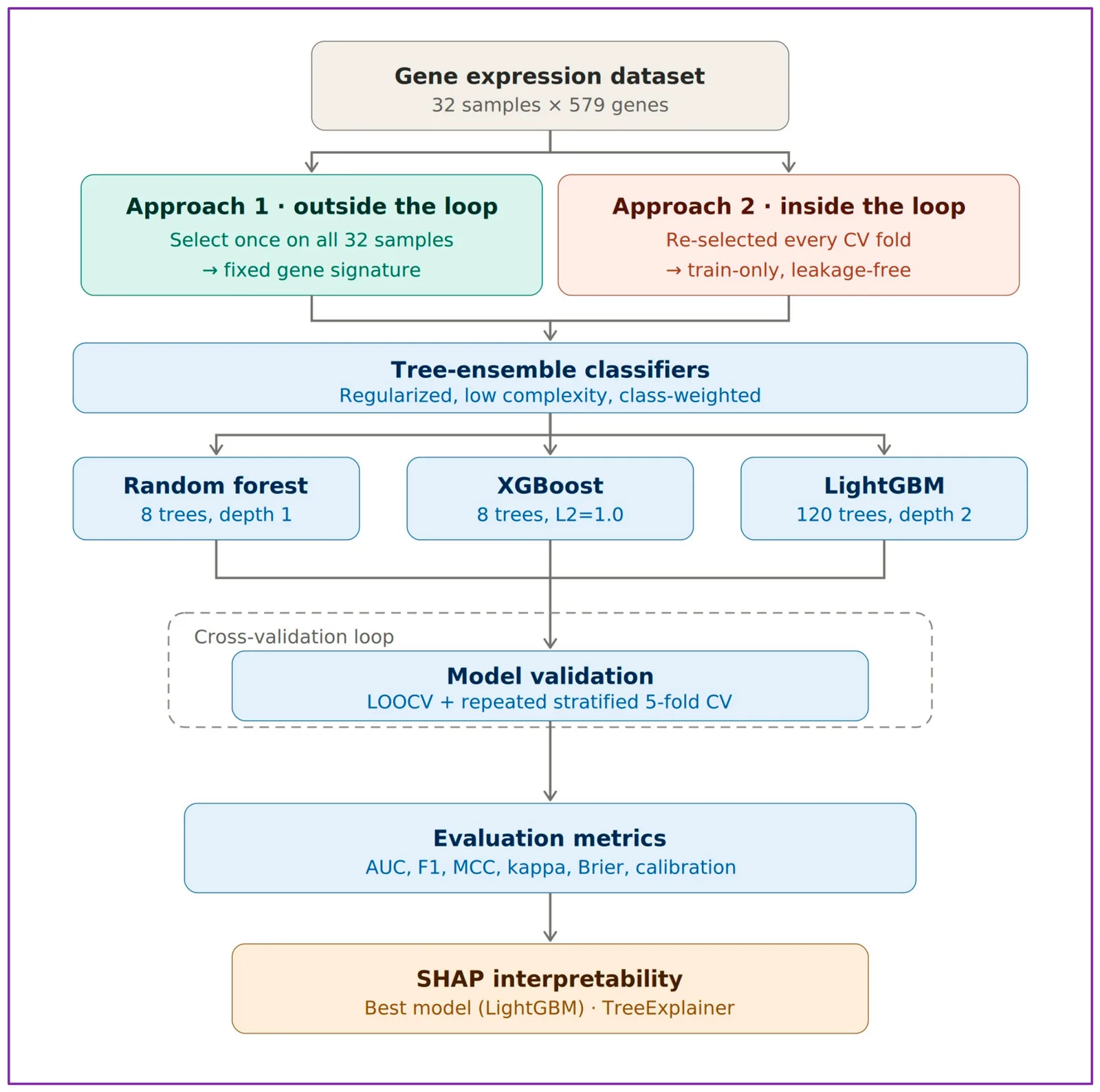
Overview of the ML workflow used in the study. The workflow illustrates the two feature-selection frameworks, the evaluated tree-based ensemble classifiers, the cross-validation strategy, the performance metrics, and SHAP-based model interpretability.

The overall ML workflow used in this study is summarized in Figure 1. The pipeline includes the two feature-selection frameworks, classifier development, leave-one-out cross-validation (LOOCV), performance evaluation, and SHAP-based model interpretability.

#### 2.2.1. Feature Selection Strategy

To identify discriminative genes within the high-dimensional expression space, we applied Elastic Net-regularized logistic regression. Elastic Net combines the L1 penalty of the least absolute shrinkage and selection operator (LASSO) with the L2 ridge penalty, thereby integrating the sparsity-inducing variable-selection property of LASSO with the improved stability of ridge regression in the presence of correlated predictors. The mixing parameter, corresponding to the L1 ratio, was fixed a priori at α = 0.7 and was not optimized within the present analysis. All gene expression variables were standardized by z-transformation before feature selection. Elastic Net regularization was used to obtain a sparse predictor set, and genes with non-zero coefficients were retained for subsequent classifier development. Feature selection was implemented using the LogisticRegression estimator in scikit-learn with penalty = ‘elasticnet’ and the SAGA solver; these settings were explicitly specified rather than left at their software defaults. The inverse regularization-strength parameter (C) was sequentially evaluated over a predefined logarithmic grid of 40 values ranging from 0.01 to 10. Rather than selecting C according to classification performance, the first C value yielding at least the prespecified target of 40 non-zero coefficients was retained. No separate cross-validation procedure was used to optimize C. In the LEFS framework, this procedure was repeated independently within each LOOCV training fold using only the corresponding training data. Feature selection was implemented under two complementary analytical frameworks. The key distinction between these frameworks was the timing of feature selection relative to model development. In the first framework, feature selection was performed once on the full dataset as a separate preprocessing step prior to classification, and the resulting fixed gene set was subsequently used for all downstream modeling and interpretability analyses. In the second framework, feature selection was not treated as a separate preliminary step; instead, it was embedded within the cross-validation procedure and repeated independently in each fold. Accordingly, the first framework was used to define a fixed and interpretable gene set, whereas the second framework was used to evaluate classifier performance when feature selection was repeated within each resampling iteration.

#### 2.2.2. Full-Dataset Feature Selection (FDFS)

In the first framework, feature selection was treated as a preprocessing step that was fully separated from the classification stage and performed before model fitting. Elastic Net regularization was applied once to the complete dataset (32 samples), and genes with coefficients shrunk to zero were excluded. Only genes with non-zero coefficients were retained, thereby defining a fixed gene set. This selection step was performed a single time, and the resulting gene set was used as an invariant input for all subsequent analyses. As a result, all classification models and interpretability analyses were conducted on the same reduced feature space, ensuring direct comparability across models. After feature selection, classification models were trained using only the selected genes, and model performance was assessed using the resampling strategy described in Section 2.2.5. SHAP-based interpretability analyses were also conducted on this same fixed gene set. Because the feature selection step in the FDFS framework leveraged information from the full dataset before model training, its primary purpose was to generate a coherent and biologically interpretable gene panel and to facilitate evaluation of its relationship to disease status. Accordingly, FDFS was treated primarily as an exploratory signature-discovery framework. Because feature selection was performed using the complete dataset before LOOCV, its classification metrics were considered descriptive and were not assigned the same inferential weight as those obtained from LEFS.

#### 2.2.3. LOOCV-Embedded Feature Selection (LEFS)

In the second framework, feature selection was incorporated directly into the modeling pipeline and repeated within each LOOCV training fold. Rather than relying on a prespecified fixed gene set, the Elastic Net procedure was rerun from the beginning within each training partition. In each fold, both feature selection and standardization were performed exclusively on the training data, whereas the held-out test sample was not used at any stage of gene selection, scaling, or model fitting. Consequently, the feature set retained in a given fold was determined solely by the corresponding training subset, and the test sample had no influence on the selection process. The LEFS framework was therefore intended to assess the robustness of the selected signature under repeated data partitioning and to evaluate model discrimination under a more stringent validation framework. LEFS was therefore considered the principal framework for sample-level internal performance evaluation, because feature selection was repeated within each LOOCV training fold without using the held-out measurement.

#### 2.2.4. Classifier Specifications

Using the selected genes, we evaluated three tree-based ensemble classifiers: Random Forest, eXtreme Gradient Boosting (XGBoost), and Light Gradient Boosting Machine (LightGBM). Given the limited sample size, all models were configured with constrained complexity to reduce overfitting; shallow trees, a limited number of estimators, subsampling strategies, and L2 regularization were preferentially used. Class imbalance (22 COVID-19 samples vs. 10 control samples) was addressed by reporting performance metrics that are informative under imbalanced classification settings. The classifier specifications were implemented as fixed, deliberately conservative configurations rather than being selected through data-driven hyperparameter optimization. No grid search, random search, Bayesian optimization, or other performance-driven hyperparameter-tuning procedure was used to determine the reported classifier settings. The repeated stratified cross-validation analysis was used only to characterize the performance variability of these fixed configurations and was not used for hyperparameter selection. This strategy was chosen to limit model-selection flexibility in view of the extremely small effective biological sample size. The specific conservative configurations were informed by preliminary experimentation with the current dataset rather than being defined entirely a priori.

The Random Forest model was specified as an ensemble of shallow trees (number of trees = 8, maximum depth = 1, minimum samples per leaf = 2). At each split, the number of candidate variables was restricted to the square root of the number of features, and class weights were set to balanced. The XGBoost model was trained using single-level trees (number of trees = 8, maximum depth = 1) with a low learning rate (0.1) and an L2 regularization coefficient of 1.0. The LightGBM model was configured with a limited-complexity tree structure (number of trees = 120, maximum depth = 2, number of leaves = 3, learning rate = 0.05) and L2 regularization of 2.0. In addition, row subsampling (subsample = 0.8) and column subsampling (colsample = 0.5) were applied to increase model diversity and further constrain overfitting. A fixed random seed was used throughout to support computational repeatability.

#### 2.2.5. Sample-Level LOOCV Evaluation

Because of the small sample size, model performance was evaluated using cross-validation rather than a single train–test split. The primary internal evaluation procedure was sample-level LOOCV, in which each sample was held out once as the test instance while the remaining samples were used for training. This procedure generated out-of-fold predicted probabilities for all samples. In the LEFS framework, the feature selection step was embedded within the corresponding resampling procedure as described above. Thus, model fitting, feature selection, and scaling were repeated within each training fold before evaluation on the held-out test sample. Because the COVID-19 arm contained repeated longitudinal measurements, sample-level LOOCV did not enforce participant-level separation; the resulting predictions were therefore interpreted as internal measurement-level evaluation rather than independent participant-level validation.

#### 2.2.6. Participant-Aware Sensitivity Analysis

To directly address the longitudinal structure of the COVID-19 data, an additional participant-aware sensitivity analysis was performed. A leave-one-COVID-participant-out outer validation strategy was used, resulting in three outer folds. In each fold, all longitudinal measurements belonging to one COVID-19 participant were excluded from the training data and evaluated together as held-out observations. Healthy-control participants were also assigned to the outer test folds at the participant level, with the 10 controls distributed across the three folds, ensuring that no participant contributed observations to both the training and test sets within a given outer fold.

All data-dependent preprocessing steps were repeated independently within each outer training set. Specifically, standardization parameters were estimated using only the training data, and Elastic Net feature selection was performed exclusively within the training participants. The Elastic Net mixing parameter was maintained at an L1 ratio of 0.7, whereas the inverse regularization-strength parameter (C) was tuned separately within each outer training set using participant-aware inner resampling. Because one of the three COVID-19 participants was held out in each outer validation fold, two COVID-19 participants remained in the corresponding outer training set. Accordingly, a two-fold participant-aware inner resampling procedure was used, with one of these two COVID-19 participants assigned to validation in each inner fold together with an approximately equal subset of the available healthy controls, thereby maintaining participant separation between the inner-training and inner-validation partitions. A predefined grid of 17 logarithmically spaced C values ranging from 0.01 to 100 was evaluated. Candidate C values were compared using mean balanced accuracy across the two inner folds, and the value with the highest mean balanced accuracy was selected, with ties resolved in favor of the smaller C value. The selected C was then used to refit Elastic Net on the complete corresponding outer training set, and genes with non-zero coefficients were retained as predictors for Random Forest, XGBoost, and LightGBM classifiers. This participant-aware tuning procedure differed from the FDFS and LEFS analyses, in which C was not optimized according to classification performance but was selected from a predefined regularization path according to the prespecified target number of non-zero coefficients. Classifier specifications were kept consistent with those used in the primary analysis. Predicted probabilities obtained from the three mutually exclusive outer test folds were pooled to calculate participant-aware out-of-fold performance metrics. Because the dataset contained only three independent COVID-19 participants, this analysis was considered an exploratory participant-aware sensitivity analysis rather than definitive external or participant-level validation. Among the validation strategies used in this study, this participant-aware analysis provided the strongest available assessment of generalization to unseen participants, although its interpretation remained constrained by the availability of only three independent COVID-19 participants.

#### 2.2.7. Performance Metrics

Model performance was assessed using a comprehensive set of metrics chosen to reflect both class discrimination and class imbalance. Discriminative performance was quantified using accuracy, balanced accuracy, sensitivity, specificity, precision, negative predictive value (NPV), F1 score, Matthews correlation coefficient (MCC), and Cohen’s kappa. For threshold-dependent metrics, class labels were assigned using a probability threshold of 0.50. Receiver operating characteristic (ROC) curves were generated to visualize model discrimination. Probability-based discrimination was evaluated using the area under the ROC curve (ROC-AUC) and the area under the precision–recall curve (PR-AUC). Probability-prediction error was assessed using the Brier score and log loss, and calibration curves were generated to provide a descriptive visualization of agreement between predicted probabilities and observed outcomes. Conventional confidence intervals were not reported for the sample-level performance metrics because the 32 measurement-level profiles do not represent 32 independent biological observations and only three independent COVID-19 participants were available. Under this repeated-measures structure, conventional interval estimates based on measurement-level observations could convey misleading precision. Therefore, the reported performance metrics were interpreted as exploratory, dataset-specific point estimates rather than population-level estimates.

#### 2.2.8. SHAP-Based Model Interpretation

To examine gene-level contributions within the LightGBM framework, we applied SHAP. SHAP analyses were performed separately for the FDFS and LEFS frameworks. For FDFS, the fixed gene set selected on the full dataset was used. For the exploratory SHAP analysis associated with LEFS, no single fold-specific LEFS feature set was used. Instead, after completion of the LEFS cross-validation procedure, Elastic Net feature selection was rerun once on the complete dataset using the same predefined feature-selection procedure (L1 ratio = 0.7 and the predefined C path), and the resulting full-dataset Elastic Net-selected gene set was used as input to the exploratory LightGBM model. For each framework, a separate LightGBM model was fitted to the full dataset solely for exploratory interpretability using a higher-capacity configuration (number of trees = 300, maximum depth = 3, number of leaves = 7), and SHAP values were computed using the TreeExplainer (SHAP, version 0.52.0) algorithm. Given the limited sample size, strongly regularized, low-complexity models were used for cross-validated performance evaluation to mitigate overfitting. In contrast, the higher-capacity LightGBM models were used only to characterize feature-contribution patterns within the selected gene sets and were not used to generate the reported predictive performance estimates. Accordingly, the resulting SHAP values describe feature contributions to these separately fitted exploratory models rather than to the predictions generated during LOOCV. Gene-level contributions were ranked according to the mean absolute SHAP value. The interpretability results were presented using two complementary visualizations: a beeswarm summary plot showing the direction and distribution of gene-level contributions for the most influential features, and a bar plot summarizing overall feature importance based on mean absolute SHAP values. For descriptive directional interpretation, normalized log2 expression values of the top SHAP-ranked genes were summarized separately for COVID-19 and healthy-control profiles. Mean differences were calculated as COVID-19 minus healthy control, and approximate fold changes were calculated as 2^(mean difference)^. No participant-level inferential testing was performed.

### 2.3. Software Environment

All analyses were performed in Python (version 3.12). Feature selection and ML models were implemented using scikit-learn (version 1.6.1), XGBoost (version 3.3.0), and LightGBM (version 4.6.0). Model interpretability analyses were conducted using SHAP (version 0.52.0). Data processing and visualization were performed using pandas (version 2.2.2), NumPy (version 2.0.2), and Matplotlib (version 3.10.0).

### 2.4. Study Protocol, and Ethical Considerations

This study was a secondary analysis of previously collected, publicly available whole-blood transcriptomic data from the E-MTAB-8871 dataset. The protocol for the present secondary analysis was approved by the Inonu University Scientific Research Ethics Committee for Health Sciences on 28 July 2026 (approval no. 10828). The study was conducted in accordance with the ethical principles of the Declaration of Helsinki, including its 2024 revision, and with applicable institutional and national regulations governing research involving human participants [32]. No additional participants were recruited, no clinical intervention or direct interaction with the original participants occurred, and the investigators did not access personally identifying information. Because the analysis was restricted to publicly available data without personally identifying information, no additional informed consent was obtained for the present secondary analysis. The study was reported in accordance with the applicable recommendations of the Strengthening the Reporting of Observational Studies in Epidemiology (STROBE) statement [33].

## 3. Results

### 3.1. Overview of the FDFS and LEFS Frameworks

Feature selection and classification results are presented separately for the two analytical frameworks defined in Section 2: the FDFS framework, in which feature selection was performed once on the full dataset before model training, and the LEFS framework, in which feature selection was embedded within the cross-validation procedure and repeated in each fold. In both frameworks, the performance of three classifiers—Random Forest, XGBoost, and LightGBM—was evaluated using sample-level LOOCV. Discriminative ability was examined using ROC curves, probability-prediction patterns were summarized using Brier scores, log loss, and calibration plots, and the feature-contribution structure of the model with the lowest observed Brier score was explored using SHAP analysis.

### 3.2. Classification Performance in the FDFS Framework

When Elastic Net regularization was applied once to the full dataset, a fixed 40-gene set was identified. As shown in Table 1, the LOOCV classification performance of the three models built on this fixed gene set was uniformly high. Within this dataset, LightGBM correctly classified all 32 measurement-level profiles (32/32) and yielded values of 1.000 across all reported sample-level discrimination metrics, including accuracy, balanced accuracy, sensitivity, specificity, precision, F1 score, MCC, Cohen’s kappa, ROC-AUC, and PR-AUC. LightGBM also showed the lowest Brier score (0.005), indicating the most favorable probability-prediction error among the evaluated models. However, because these 32 profiles were derived from only 13 unique participants, including three longitudinally sampled COVID-19 participants, these findings should be interpreted as exploratory, dataset-specific estimates of internal measurement-level discrimination rather than evidence of generalizable classification performance.

Also in Table 1, Random Forest and XGBoost each misclassified one profile (31/32; accuracy = 0.969). Both models retained high sensitivity (1.000) and specificity (0.900), but their probability-prediction profiles differed substantially. Random Forest achieved a perfect ROC-AUC of 1.000 with a comparatively low Brier score of 0.017, whereas XGBoost yielded a lower ROC-AUC of 0.900 together with the least favorable probability-prediction performance among the three models (Brier score = 0.087; log loss = 0.331).

**Figure 2:**
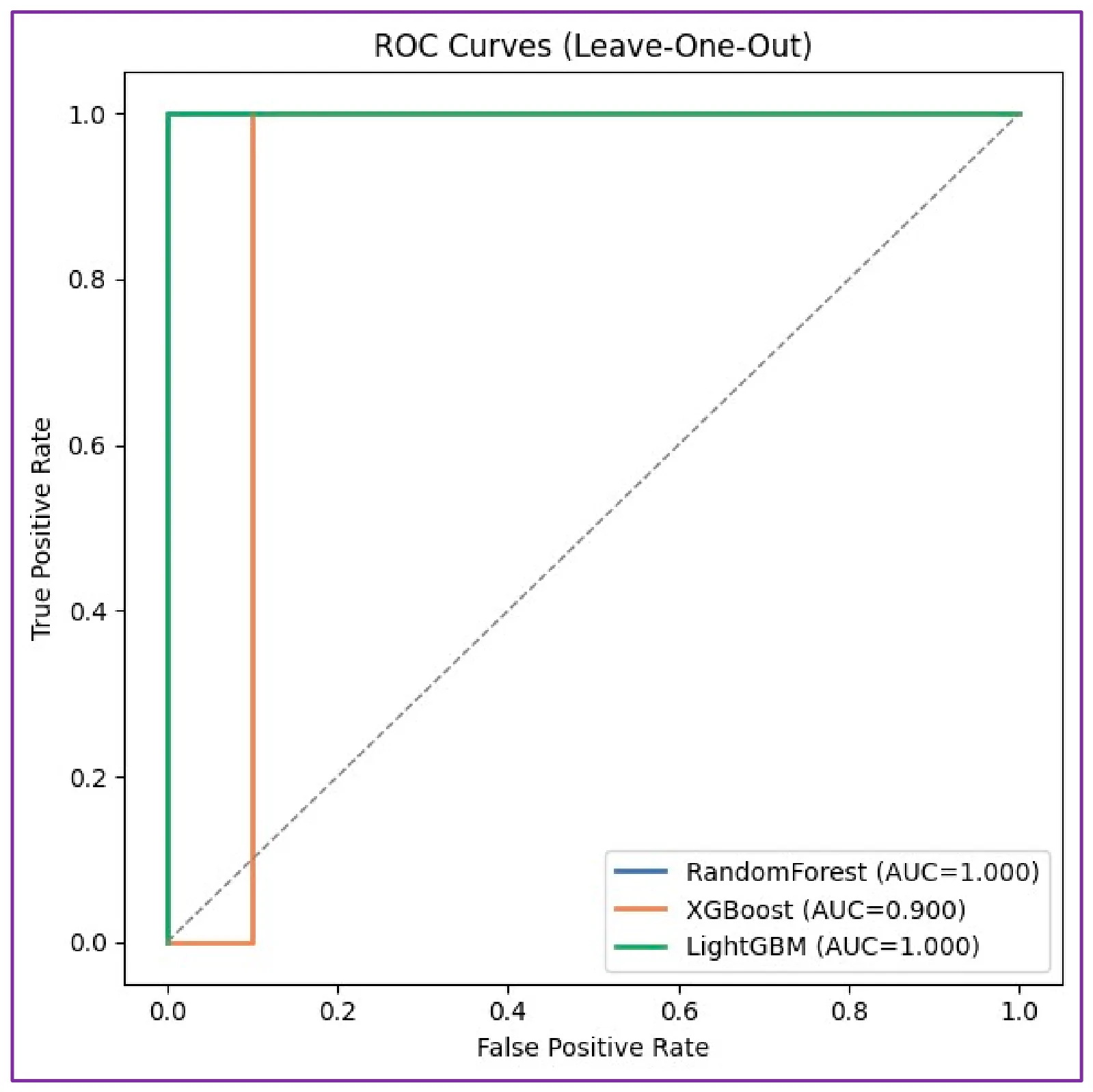
ROC curves for FDFS framework under leave-one-out cross-validation. The Random Forest and LightGBM ROC curves overlap because they show identical ROC performance (AUC = 1.000). The dashed diagonal line represents chance-level (no-discrimination) performance.

**Figure 3:**
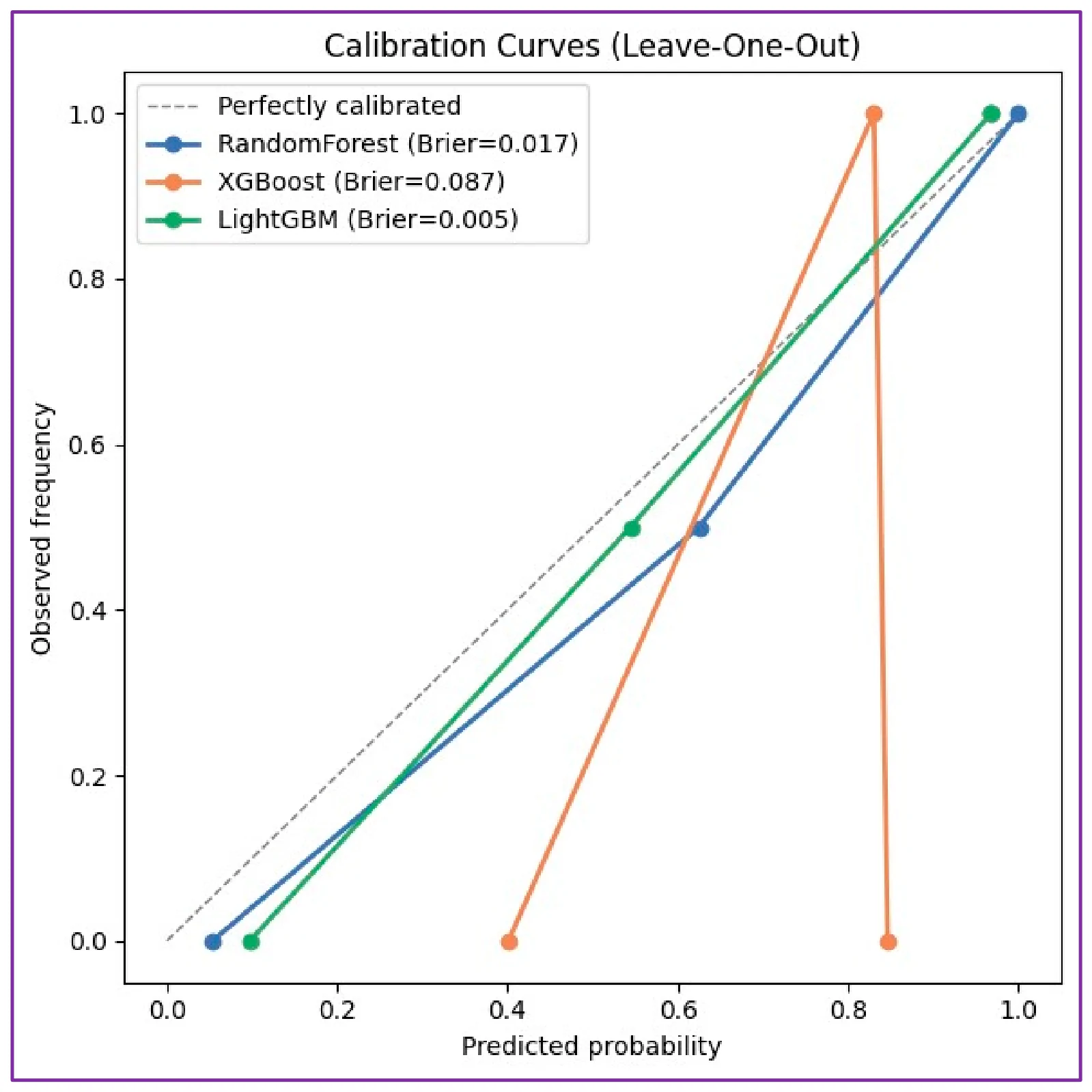
Calibration curves for FDFS framework under leave-one-out cross-validation.

These results are visually supported by the discrimination and calibration plots. As shown in Figure 2, the ROC curves demonstrate perfect or near-perfect measurement-level discrimination, with Random Forest and LightGBM achieving ROC-AUC values of 1.000. Likewise, Figure 3 shows that LightGBM displayed the closest descriptive agreement with the ideal calibration line and showed the most favorable observed Brier score and calibration-plot pattern. Given the small number of measurement-level profiles (*n* = 32), the calibration curves should be interpreted as descriptive visualizations rather than as robust evidence of model calibration. Formal assessment of calibration was not considered feasible with this sample size; therefore, the Brier score was retained as a descriptive measure of overall probabilistic prediction error.

These FDFS performance estimates should be interpreted descriptively within a signature-discovery framework, because the fixed gene set was selected using the complete dataset before LOOCV. Principal sample-level internal performance estimates are therefore reported from the LEFS framework below.

### 3.3. SHAP-Based Interpretation in the FDFS Framework

**Figure 4:**
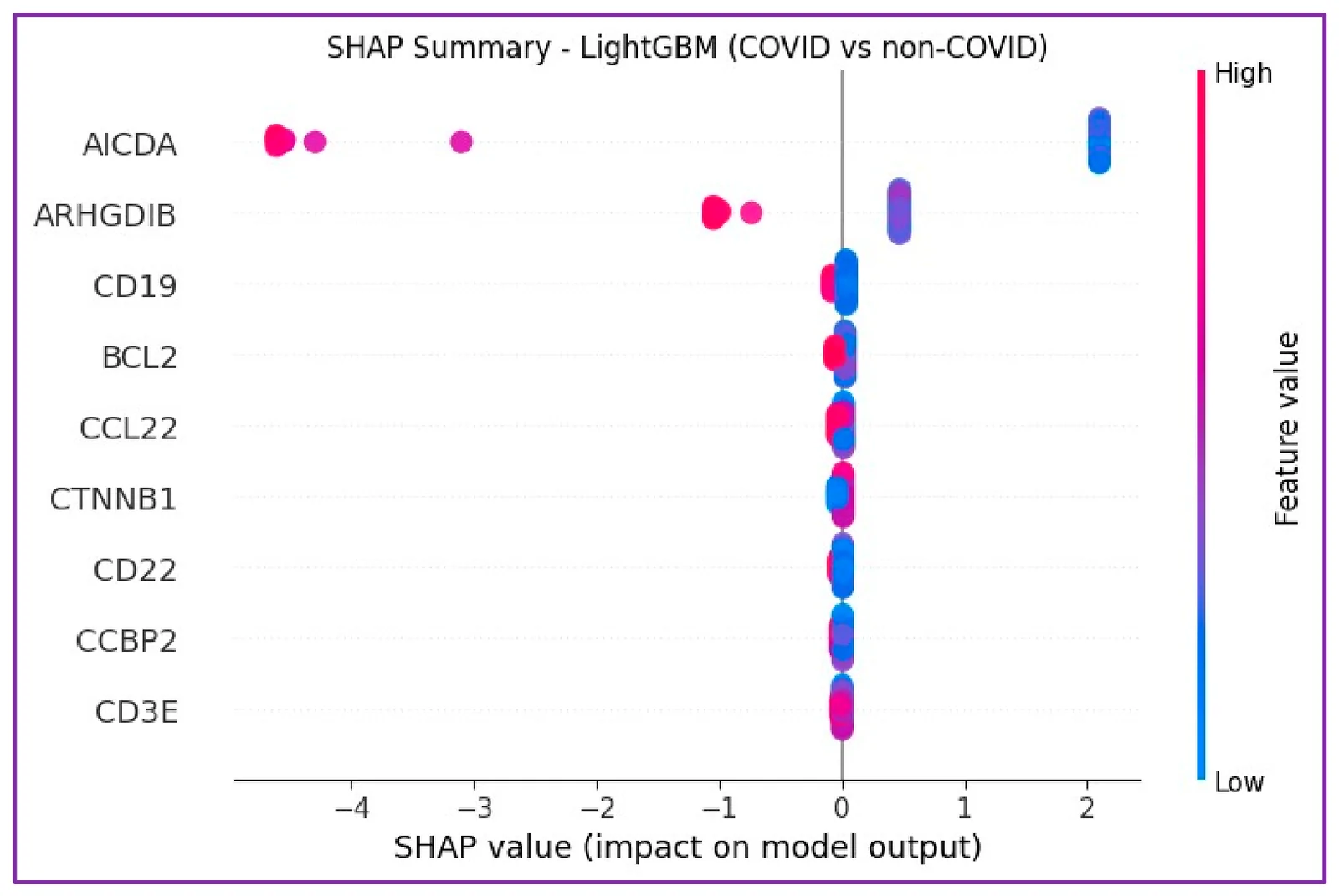
LightGBM SHAP summary (beeswarm) plot for FDFS framework (top 9 genes).

**Figure 5:**
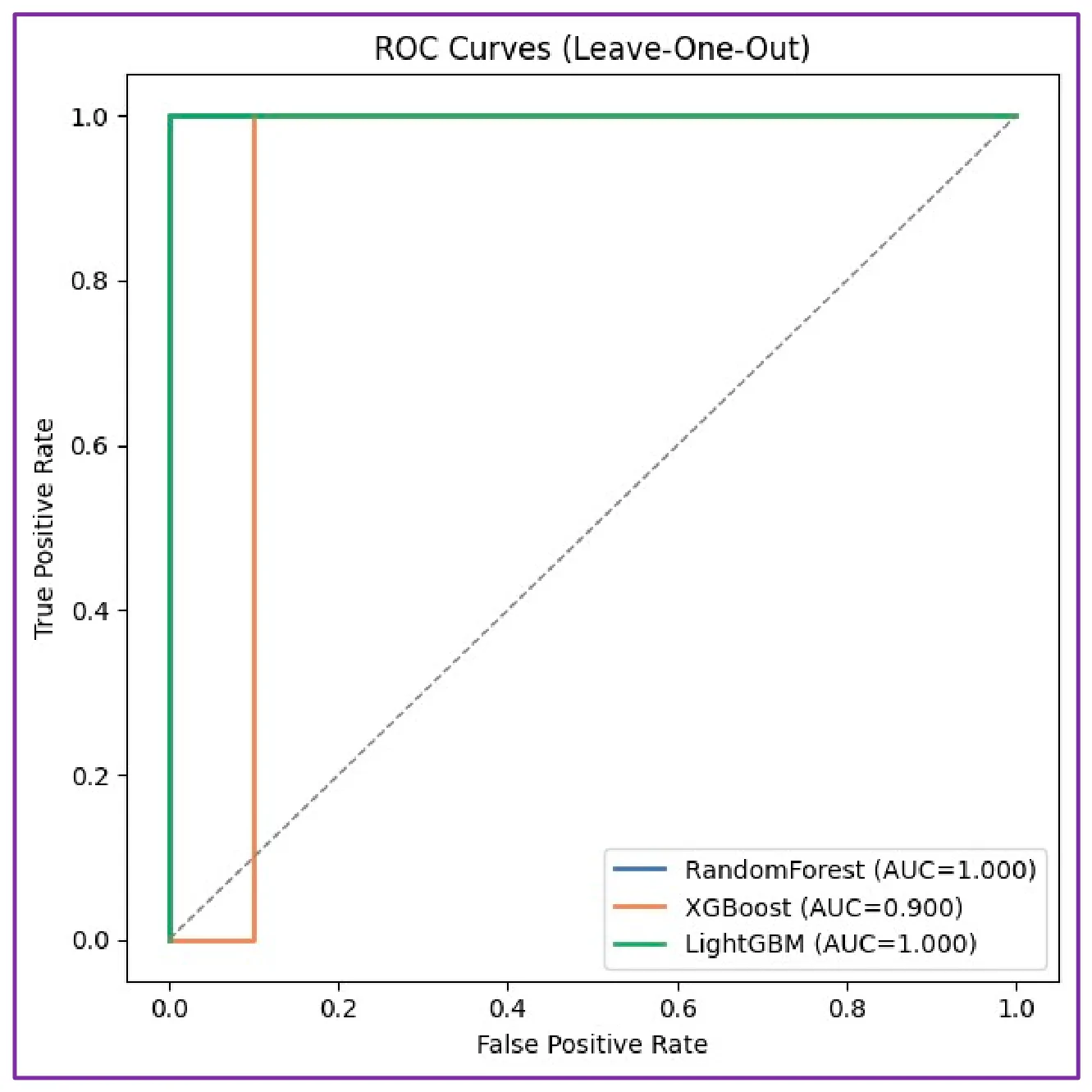
ROC curves for LEFS framework under leave-one-out cross-validation. The dashed diagonal line represents chance-level (no-discrimination) performance..

Because LightGBM combined the highest discriminative performance with the lowest Brier score, a separate exploratory LightGBM model was fitted to characterize feature contributions using SHAP analysis. As summarized in Table 2, ranking features by mean absolute SHAP value revealed a strongly concentrated contribution pattern. *AICDA* was the dominant contributor (mean |SHAP| = 2.824; LightGBM importance = 236), followed by *ARHGDIB* (mean |SHAP| = 0.638; importance = 50). The contribution of the remaining genes was markedly smaller, with *CD19*, *BCL2*, *CCL22*, *CTNNB1*, *CD22*, *CCBP2*, and *CD3E* showing only modest incremental influence on model output. This distribution is also evident in the SHAP visualizations. As illustrated in Figure 4, the beeswarm summary plot showed a clear directional separation for *AICDA*, with high and low expression values corresponding to opposite directions of contribution to the predicted class. In parallel, Figure 5 confirms that *AICDA* and *ARHGDIB* dominated the overall importance structure, whereas the remaining genes contributed only limited incremental information. Taken together, Table 2 and Figure 4 and Figure 5 show that the SHAP contribution pattern was concentrated predominantly in a small subset of model-prioritized features rather than distributed diffusely across the full feature set.

### 3.4. Classification Performance in the LEFS Framework

Under the feature-selection- and standardization-leakage-reduced LEFS framework, in which Elastic Net feature selection was repeated within each cross-validation fold, the number of selected genes showed limited fold-to-fold variation, with a mean of 42 genes per fold (range, 40–47). To determine whether this numerical consistency also reflected stability in gene identity, feature-selection stability was quantified across all 32 LEFS folds. Thirty-one genes were selected in all 32 folds (100%), 36 genes were selected in at least 90% of folds, 39 genes in at least 75% of folds, and 40 genes in at least 50% of folds. Notably, *AICDA* and *ARHGDIB* were selected in all 32 folds. Across the 496 pairwise comparisons of the selected feature sets, the mean Jaccard similarity was 0.847 (median, 0.854; range, 0.635–0.979). The generalized Kuncheva stability index was also high, with a mean of 0.933 (median, 0.946; range, 0.783–1.000). These findings indicate substantial stability in the identity of the genes selected across LEFS folds, rather than merely consistency in the number of selected features. Gene-specific selection frequencies are provided in Supplementary Table S1.

**Figure 6:**
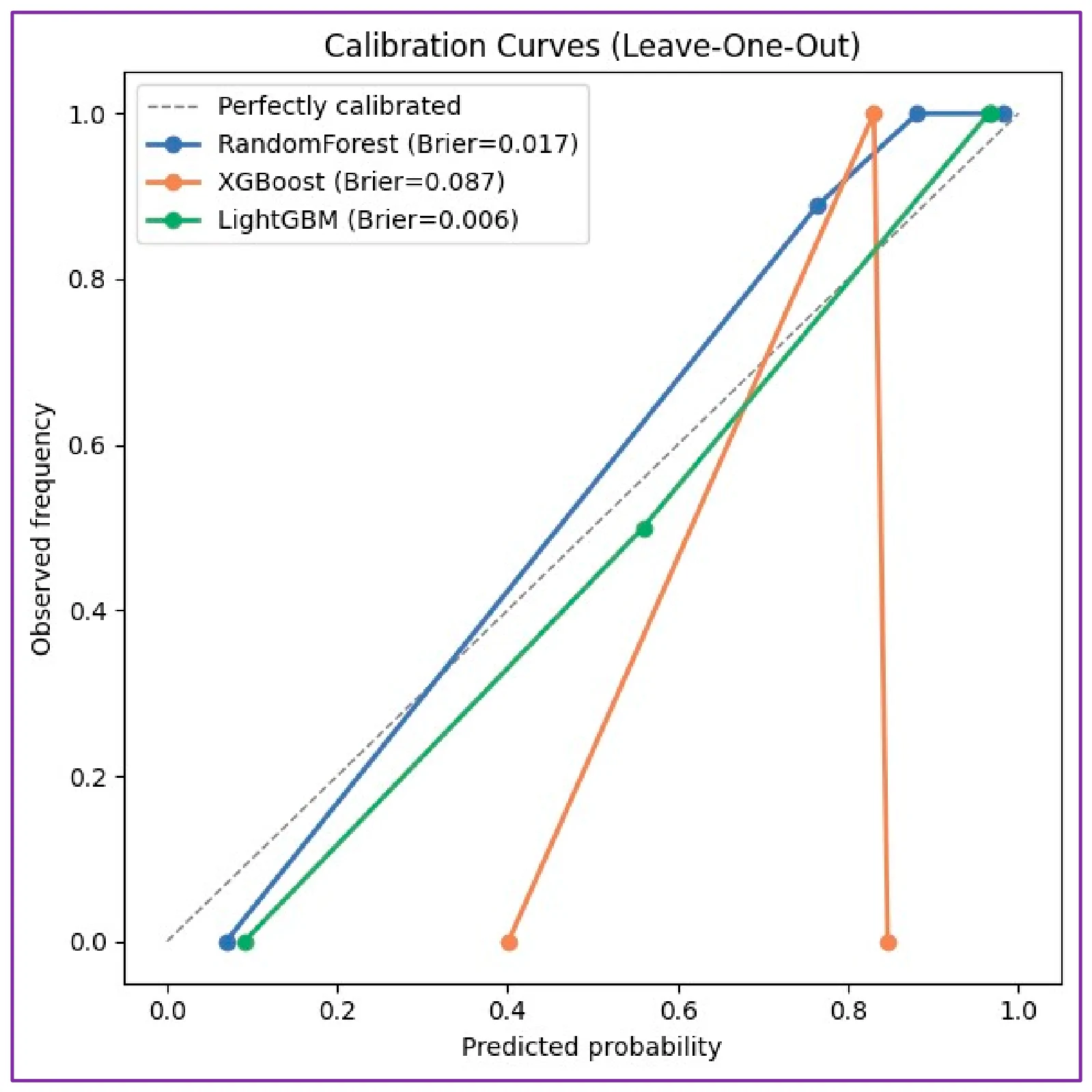
Calibration curves for LEFS framework under leave-one-out cross-validation.

When feature selection was repeated within each LOOCV training fold, classification performance remained high across all three models. As shown in Table 3, Random Forest and LightGBM both correctly classified all 32 measurement-level profiles (derived from 13 unique participants: 10 healthy controls and three longitudinally sampled COVID-19 participants) (32/32; accuracy = 1.000). Given the extremely limited number of independent biological participants, particularly the presence of only three COVID-19 participants, these results should be interpreted as exploratory and dataset-specific estimates of internal measurement-level discrimination rather than as evidence of generalizable classification performance. According to Table 3, Random Forest achieved values of 1.000 across all reported sample-level discrimination metrics and a Brier score of 0.017, whereas LightGBM likewise maintained perfect measurement-level discrimination with a lower Brier score of 0.006, indicating the lowest observed probability-error score among the evaluated models. XGBoost again misclassified one profile (31/32; accuracy = 0.969), with sensitivity of 1.000, specificity of 0.900, ROC-AUC of 0.900, and a Brier score of 0.087. The graphical results were consistent with these tabulated findings. As shown in Figure 5, discriminative performance remained strong even after feature selection was fully embedded within the LOOCV loop, with Random Forest and LightGBM both achieving ROC-AUC values of 1.000. Similarly, Figure 6 demonstrates that LightGBM again showed the most favorable descriptive calibration-plot pattern and the lowest Brier score among the evaluated models. Given the small number of measurement-level profiles (*n* = 32), the calibration curves should be interpreted as descriptive visualizations rather than as robust evidence of model calibration. Formal assessment of calibration was not considered feasible with this sample size; therefore, the Brier score was retained as a descriptive measure of overall probabilistic prediction error.

LEFS was therefore considered the principal framework for sample-level internal performance evaluation, because feature selection and standardization were repeated within each LOOCV training fold without using the held-out measurement.

### 3.5. Exploratory SHAP Interpretation Using the Full-Dataset Elastic Net-Selected Feature Set

**Figure 7:**
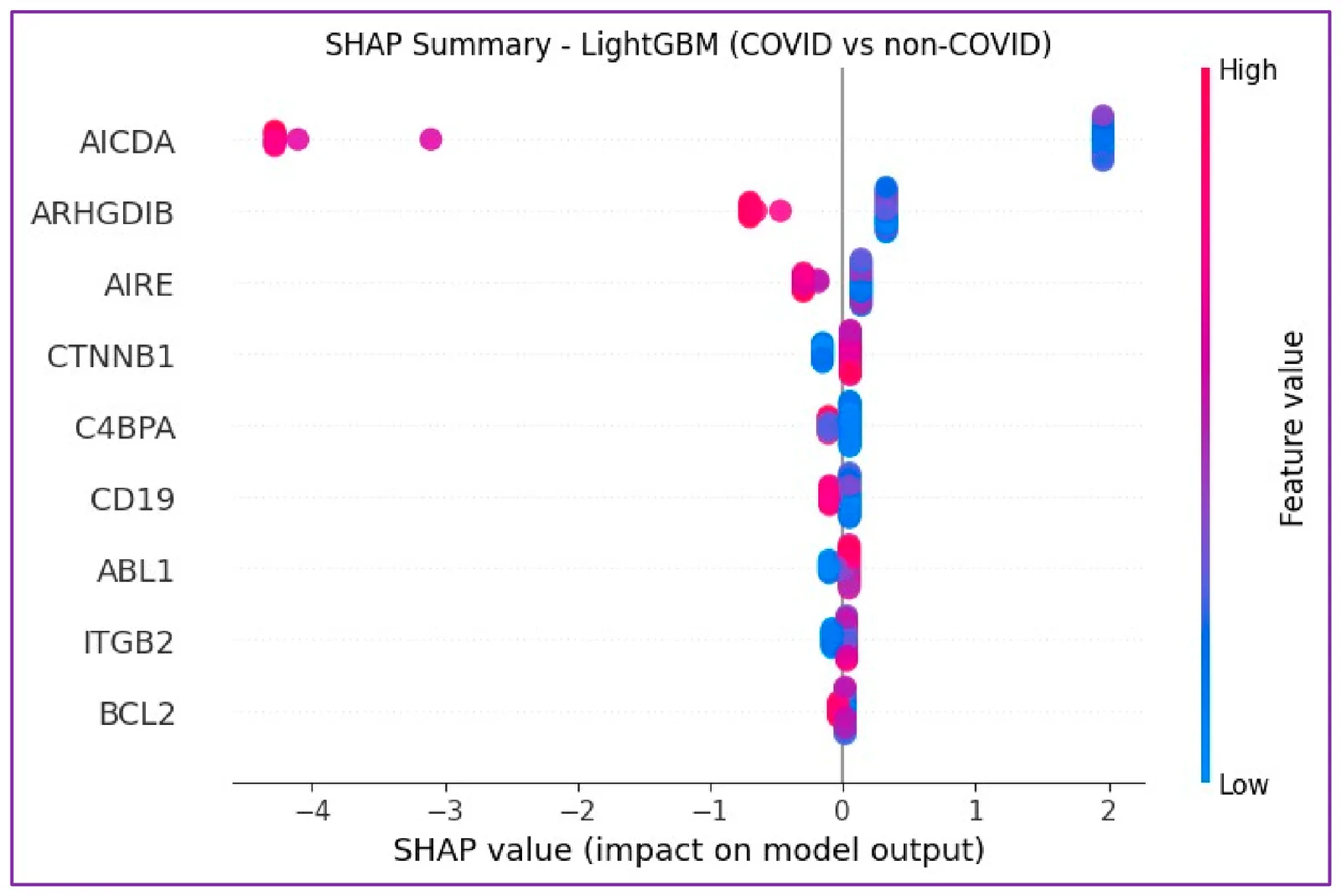
LightGBM SHAP summary (beeswarm) plot based on the full-dataset Elastic Net-selected feature set (top 9 genes).

**Figure 8:**
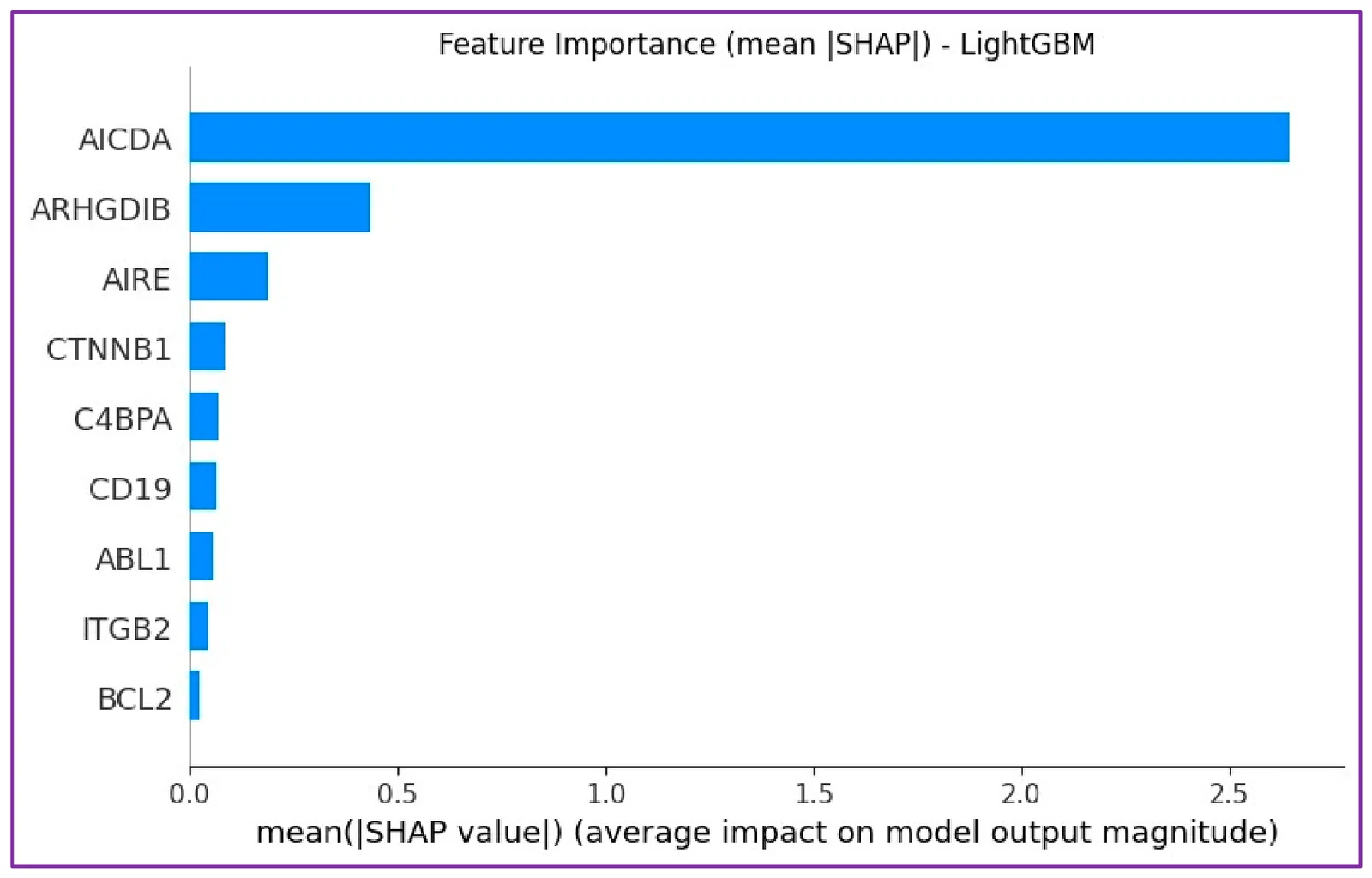
Mean SHAP feature importance plot for the LightGBM model based on the full-dataset Elastic Net-selected feature set (top 9 genes).

The exploratory SHAP analysis using the full-dataset Elastic Net-selected feature set is summarized in Table 4 and visualized in Figure 7 and Figure 8. As shown in Table 4, the contribution profile again revealed a strongly dominant role for *AICDA* (mean |SHAP| = 2.642; LightGBM importance = 225), followed by *ARHGDIB* (mean |SHAP| = 0.435; importance = 36). Additional contributors included *AIRE* (0.186), *CTNNB1* (0.086), *C4BPA* (0.069), *CD19* (0.065), *ABL1* (0.054), *ITGB2* (0.045), *BCL2* (0.021), and *CCR5* (0.019). This ranking pattern is mirrored in the SHAP plots. Figure 7 shows that *AICDA* had the broadest and most influential SHAP value distribution, while Figure 8 confirms the marked separation in mean absolute SHAP importance between the top two genes and the remaining variables. Although the secondary features differed slightly from those observed in the FDFS exploratory SHAP analysis, *AICDA* and *ARHGDIB* remained the two highest-ranked features in the present full-dataset Elastic Net-based exploratory SHAP model. This broadly similar ranking indicates within-dataset consistency in model-prioritized features, but it should not be interpreted as evidence of consistent prioritization across LEFS folds, independent participants, or external cohorts.

### 3.6. Participant-Aware Sensitivity Analysis Results

To evaluate whether the high measurement-level performance observed under sample-level LOOCV could be explained predominantly by within-participant similarity, an additional participant-aware sensitivity analysis was performed. In each of three outer folds, all longitudinal measurements from one COVID-19 participant were withheld simultaneously, together with a participant-level subset of healthy controls. Standardization, Elastic Net feature selection, and classifier fitting were repeated using only the remaining training participants.

LightGBM maintained perfect discrimination under this more stringent validation strategy, correctly classifying all 32 held-out profiles (accuracy = 1.000, sensitivity = 1.000, specificity = 1.000, ROC-AUC = 1.000, PR-AUC = 1.000), with a Brier score of 0.019. Importantly, perfect classification was observed separately when Case 1, Case 2, and Case 3 were excluded from model development, with 13/13, 12/12, and 7/7 held-out observations correctly classified, respectively. Random Forest also showed high participant-aware performance, with an accuracy of 0.969, sensitivity of 1.000, specificity of 0.900, ROC-AUC of 0.998, PR-AUC of 0.998, and Brier score of 0.049. XGBoost showed comparatively lower performance, with an accuracy of 0.938, sensitivity of 0.955, specificity of 0.900, ROC-AUC of 0.875, PR-AUC of 0.904, and Brier score of 0.103 (Table 5).

Elastic Net feature selection was repeated independently within each outer training fold and retained 54, 29, and 25 genes when Case 1, Case 2, and Case 3, respectively, were held out. Thus, the participant-aware results were not based on the fixed 40-gene set derived from the complete dataset. Although these findings indicate that the strong discrimination observed for LightGBM was preserved when all measurements from an individual COVID-19 participant were excluded from model development, they should be interpreted cautiously because participant-level generalizability remains constrained by the availability of only three independent COVID-19 participants.

To further investigate the robustness of the observed discrimination, an exact participant-level permutation test was performed for LightGBM. Disease labels were reassigned at the participant level while preserving all longitudinal measurements from each participant as a single unit. Because the dataset comprised 13 independent participants, including three COVID-19 participants, all 286 possible assignments of three COVID-19 labels among the 13 participants were evaluated. For each of the 286 possible participant-level label assignments, the outer validation folds were reconstructed according to the permuted labels. The three participants assigned the COVID-19 label were held out one at a time across the three outer folds, while participants assigned as controls were distributed across the corresponding test folds. All measurements belonging to a given participant were kept together, ensuring that no participant contributed observations to both training and test data within the same outer fold. The complete training procedure was repeated independently for each permuted assignment. The complete training procedure, including training-only standardization, Elastic Net feature selection, and model fitting, was repeated for each assignment. The observed participant-aware ROC-AUC was 1.000, whereas the null distribution had a mean ROC-AUC of 0.575 (median, 0.569; SD, 0.198; range, 0.122–1.000). Only 3 of the 286 possible label assignments produced a ROC-AUC equal to or greater than the observed value, yielding an exact permutation *p*-value of 0.0105. Thus, the observed discrimination exceeded that expected under participant-level random label assignment; nevertheless, given the availability of only three independent COVID-19 participants, this finding should be interpreted as supportive evidence of robustness rather than as confirmation of predictive generalizability.

### 3.7. Descriptive Expression Patterns of the Top SHAP-Ranked Genes

To clarify the direction of expression differences among the highest-contributing genes, normalized log2 expression values were summarized descriptively for COVID-19 and healthy-control profiles. *AICDA*, *ARHGDIB*, *CD19*, *BCL2*, *AIRE*, *C4BPA*, *CCL22*, *CD22*, *CCBP2*, and *CD3E* showed lower mean expression in COVID-19 profiles, whereas *CTNNB1*, *ABL1*, *ITGB2*, and *CCR5* showed higher mean expression. The corresponding descriptive means, log2 differences, approximate fold changes, and SHAP importance values are presented in Table 6. Because the COVID-19 group consisted of repeated measurements from only three participants, these comparisons were interpreted descriptively and not as participant-level differential-expression estimates.

### 3.8. Comparison of the FDFS and LEFS Frameworks

The FDFS and LEFS frameworks yielded broadly concordant measurement-level classification results. FDFS identified a fixed 40-gene set, whereas LEFS retained a mean of 42 genes per fold, with a range of 40–47. This narrow range indicates limited variation in the number of selected features across LOOCV folds, although it does not by itself demonstrate stability of the exact gene composition. Across the two frameworks, classification accuracy ranged from 0.969 to 1.000. LightGBM correctly classified all 32 measurement-level profiles (derived from 13 unique participants: 10 healthy controls and three longitudinally sampled COVID-19 participants) in both frameworks and produced the lowest observed Brier score, with values of 0.005 for FDFS and 0.006 for LEFS. Random Forest accuracy increased from 0.969 under FDFS to 1.000 under LEFS, whereas XGBoost achieved an accuracy of 0.969 in both frameworks. The corresponding SHAP analyses also showed similar leading features, with *AICDA* and *ARHGDIB* ranking as the two most influential features in both frameworks. Overall, the concordant model performance and feature-ranking patterns indicate within-dataset consistency across the two feature-selection frameworks. However, these findings do not establish stability of the complete selected gene set or performance in previously unseen participants, particularly because the COVID-19 group consisted of repeated measurements from only three individuals. Importantly, the three analytical frameworks should not be assigned equivalent inferential weight. FDFS should be regarded primarily as an exploratory signature-discovery framework because its fixed predictor set was selected using the complete dataset before LOOCV, allowing the held-out measurement to influence the selected feature space. Consequently, its classification metrics are descriptive and may be optimistically biased. LEFS provides the principal sample-level internal validation results because feature selection and standardization were repeated within each LOOCV training fold without using the held-out measurement. The participant-aware sensitivity analysis provides the strongest available assessment of generalization in the present dataset because all longitudinal measurements from each held-out COVID-19 participant were excluded from model development in turn. Nevertheless, given that only three independent COVID-19 participants were available, even the participant-aware results should be considered exploratory and should not be interpreted as definitive evidence of generalizability to independent populations.

## 4. Discussion

In this exploratory secondary analysis of a publicly available longitudinal whole-blood transcriptomic dataset, we evaluated whether NanoString Human Immunology Panel-based whole-blood transcriptomic profiles could discriminate COVID-19 measurements from healthy-control measurements and whether the timing of feature selection influenced the performance of tree-based ensemble classifiers. Across both analytical frameworks, classification performance was uniformly high, with LightGBM showing the most favorable overall profile, particularly with respect to the observed Brier score and log loss. In addition, the two frameworks converged on a similar interpretability pattern, with *AICDA* and *ARHGDIB* consistently emerging as the most influential contributors to model output by a wide margin. Taken together, these findings provide proof-of-concept evidence that targeted immune-response transcriptomic profiles contain a compact classification signal in this dataset and that the FDFS and LEFS frameworks yield broadly concordant internal results. Because the COVID-19 profiles included repeated longitudinal measurements from only three participants, this performance reflects measurement-level discrimination and requires confirmation through independent participant-level external validation.

These findings are broadly consistent with prior transcriptomic and immunological studies showing that blood-based host-response signatures can capture clinically relevant aspects of SARS-CoV-2 infection. Earlier studies identified dysregulated interferon responses, inflammatory cytokine signaling, altered myeloid-cell programs, and changes in lymphocyte activation and differentiation as central features of COVID-19 immunobiology [6,10,12,17]. Other host-response transcriptome studies have shown that whole-blood transcriptional profiles can stratify patients according to disease severity [13,18], whereas cross-tissue transcriptomic analyses have investigated infection-status classification [14]. Several ML pipelines have also been used for patient classification, severity stratification, or blood biomarker discovery [9,22,23]. Within that context, the present study adds a focused proof-of-concept using a targeted NanoString panel rather than genome-wide RNA-seq and shows that strong within-dataset discrimination can be observed even in a compact, immune-focused expression space. The present analysis also differs from many previous studies by directly comparing the FDFS and LEFS frameworks, thereby addressing not only classification performance but also the methodological consequences of when feature selection is performed.

The use of a targeted NanoString panel is itself relevant to the interpretation of the results. Compared with genome-wide RNA-seq, targeted digital transcript counting offers a more constrained representation of the host response [21], and targeted immune panels may offer translational relevance because the assayed genes are already enriched for immunological relevance [20]. NanoString-based profiling has been applied to COVID-19 whole-blood transcript profiling and longitudinal immune-response characterization [16,19]. In parallel, focused targeted transcript panels have been proposed as practical tools for translational immune profiling and biomarker development [20]. The present findings are compatible with that rationale and suggest that a restricted immune-gene panel may still preserve sufficient biological information for strong within-dataset measurement-level classification in a high-dimensional but small-sample setting. This is translationally relevant because focused panel development seeks to move from broad discovery profiles toward smaller, predefined transcript sets [20]; however, the present study did not evaluate prospective clinical implementation or diagnostic utility.

A second notable aspect of the present study is the comparison between FDFS and LEFS as complementary analytical frameworks. In the present dataset, embedding feature selection within cross-validation did not lead to a meaningful loss of performance. Rather, classification performance remained broadly concordant across the two frameworks, and LightGBM retained the most favorable overall profile in both approaches. Numerically, LightGBM showed slightly better calibration in the FDFS framework than in the LEFS framework, whereas Random Forest improved from 0.969 to 1.000 accuracy under the LEFS framework; taken together, these findings do not indicate a clear overall winner between the two frameworks, but instead show that the main discriminative performance remained broadly consistent when feature selection and standardization were re-estimated within each LOOCV training fold. The recurrence of the same dominant genes across both frameworks likewise indicates within-dataset consistency in the leading model-prioritized features, although it does not establish stability of the exact gene composition. From a methodological perspective, FDFS is best regarded as a signature-discovery and biological-interpretation framework, whereas LEFS provides a feature-selection- and standardization-leakage-reduced framework for internal performance evaluation. They should not be interpreted as competing clinical validation approaches.

At the same time, the distinction between these approaches remains methodologically important. In high-dimensional biological classification, selection of features before resampling can lead to optimistic performance estimates because the predictor set is informed by the full dataset [24,34,35], whereas repeating feature selection within each fold reduces feature-selection-related optimism in internal resampled performance estimates [24,34,35]. Therefore, in the context of the present results, the principal value of the LEFS framework is not that it outperformed the FDFS framework numerically, but that it preserved near-identical performance under a preprocessing-leakage-reduced internal evaluation framework. Nevertheless, LOOCV was performed at the sample level: when one COVID-19 measurement was held out, other longitudinal measurements from the same participant could remain in the training set. LEFS therefore reduced leakage arising from feature selection and standardization but did not provide participant-level independence. Accordingly, the comparison between the two frameworks is best understood as a demonstration of internal consistency rather than as formal evidence of external generalizability.

The gene-level interpretability results also merit discussion. Across both analytical frameworks, *AICDA* was the dominant contributor, followed by *ARHGDIB*, with all remaining genes contributing substantially smaller SHAP values. SHAP magnitude quantifies a feature’s contribution to the fitted model output; it does not establish causal importance or disease specificity and does not by itself indicate whether the corresponding gene is more highly or more weakly expressed. *AICDA* encodes activation-induced cytidine deaminase, a key enzyme involved in immunoglobulin class-switch recombination and somatic hypermutation and therefore closely linked to activated B-cell and germinal-center biology [36,37,38]. Its prominence in the current models may reflect B-cell-related variation in the whole-blood profiles represented in this dataset, rather than identifying a mechanistic driver. This interpretation is biologically plausible in light of previous studies describing marked B-cell perturbation, plasmablast expansion, and broader adaptive immune remodeling during SARS-CoV-2 infection [11,39,40,41,42,43]. *ARHGDIB* encodes Rho GDP-dissociation inhibitor beta, a hematopoietic-cell-associated regulator of Rho-family GTPase signaling [44,45,46]. Its recurrent importance in both approaches may be consistent with variation in leukocyte composition, activation, migration, or lymphocyte signaling, although this interpretation remains indirect in the absence of cell-type-resolved validation [47,48].

The descriptive expression analysis provided directional context for the SHAP findings. *AICDA* and *ARHGDIB*, the two most influential genes in both frameworks, showed lower mean expression in COVID-19 profiles than in healthy controls. The lower *AICDA* signal may be compatible with shifts across B-cell differentiation states, including lower *AICDA* expression during later plasma-cell differentiation [38,49,50]. However, this interpretation remains indirect in bulk whole-blood data and cannot be assigned to a specific cellular population. Plasmablast expansion has been repeatedly documented in COVID-19 [39,41,43], but these observations do not establish that plasmablast expansion generated the lower bulk *AICDA* signal observed in the present dataset. In addition, an independent single-cell analysis of peripheral B cells reported that *ARHGDIB* expression was downregulated during moderate and severe SARS-CoV-2 infection and increased again during convalescence [51]. This finding provides contextual support but does not demonstrate that the same cellular process produced the model association observed here. Several other B-cell-associated genes, including *CD19* and *CD22*, showed lower mean expression, whereas *CTNNB1*, *ABL1*, *ITGB2*, and *CCR5* showed higher mean expression. These descriptive patterns may reflect differences in circulating immune-cell composition, activation states, and disease timing rather than gene-specific mechanistic effects.

At the same time, these genes should not be overinterpreted as definitive mechanistic drivers of COVID-19 biology on the basis of the present analysis alone. This study was not designed as a mechanistic transcriptomics study and did not include orthogonal validation at the protein, cell-type, or functional level. Accordingly, *AICDA* and *ARHGDIB* are best regarded here as model-dependent, model-prioritized transcriptomic features that may reflect biologically meaningful variation in the analyzed whole-blood profiles and that merit further study in larger, independently sampled cohorts. The same principle applies to the secondary genes identified by SHAP, including B-cell-associated, signaling-related, and immune-regulatory transcripts. The identification of overlapping secondary features across FDFS and LEFS indicates some within-dataset consistency in model prioritization, but their exact biological roles cannot be established from this dataset alone.

### 4.1. Strengths of the Study

Several strengths of the study should be emphasized. (i) The work used a publicly available dataset with accessible raw and processed files, allowing transparent reanalysis. (ii) The study addressed a targeted whole-blood immune transcriptome generated on a platform with direct digital counting [21] and potential translational relevance [20]. (iii) The analysis explicitly compared two feature-selection frameworks rather than presenting only a single optimized classification result, an issue of recognized methodological importance in high-dimensional biomarker studies [24,25]. (iv) Model performance was characterized using multiple threshold-dependent, discrimination, and probability-based metrics rather than accuracy alone. (v) The incorporation of SHAP analysis provided a bridge between prediction and model interpretation, allowing a small number of influential, model-prioritized genes to be identified [29]. (vi) Collectively, these elements provide a transparent, hypothesis-generating methodological basis for future independent validation studies.

### 4.2. Limitations of the Study

The study also has important limitations. (i) The most substantial limitation is the structure of the underlying dataset. Although 32 expression profiles were analyzed, the 22 COVID-19 samples were derived from only three participants sampled longitudinally, whereas the control arm consisted of 10 distinct individuals. Consequently, the number of profiles substantially exceeds the number of independent biological units in the COVID-19 group. (ii) Because LOOCV was conducted at the sample level, repeated measurements from the same COVID-19 participant could contribute to training while another measurement from that participant served as the held-out observation. The resulting performance estimates therefore describe internal measurement-level discrimination rather than independent participant-level validation [52,53]. To examine this issue directly, we performed an additional participant-aware sensitivity analysis in which all longitudinal measurements from one COVID-19 participant were excluded from model development in each outer fold, with standardization, Elastic Net feature selection, and classifier fitting repeated exclusively using the remaining participants. LightGBM retained an accuracy, ROC-AUC, and PR-AUC of 1.000, with all measurement-level profiles correctly classified when each of the three COVID-19 participants was held out in turn. Random Forest similarly retained strong discrimination, whereas XGBoost showed a greater reduction in performance. These findings suggest that the observed discrimination was not entirely dependent on within-participant overlap between the training and test sets. Nevertheless, because only three independent COVID-19 participants were available, this sensitivity analysis cannot establish robust participant-level generalizability and should be regarded as supportive exploratory evidence rather than definitive validation. (iii) A related limitation is the strong *p* ≫ *n* structure of the data, which increases the risk of instability and overfitting despite the use of regularization, constrained model complexity, and LOOCV-embedded feature selection in the LEFS framework. These procedures reduce but do not eliminate model-instability and overfitting risks. Moreover, the very small number of independent COVID-19 participants precluded reliable estimation of conventional confidence intervals for model-performance metrics. Accordingly, the reported point estimates, including values of 1.000 for some performance measures, should not be interpreted as precise population-level estimates but rather as exploratory, dataset-specific measures of internal performance. (iv) The comparator group consisted exclusively of healthy controls, which is an important limitation of the present study. Consequently, the observed classification signal should not be interpreted as COVID-19-specific, as the analysis establishes discrimination only between COVID-19 and healthy-control profiles within this dataset. Whether the identified transcriptomic patterns can distinguish COVID-19 from other infectious, inflammatory, or respiratory conditions remains unknown and requires evaluation using clinically relevant disease-control groups. (v) In addition, the control group and COVID-19 group differ not only by disease status but also by longitudinal structure, and the contributions of disease status, participant identity, and sampling time cannot be fully disentangled in this small dataset. (vi) No independent external dataset was available to assess the selected features or model performance in previously unseen participants. (vii) Finally, SHAP analysis was performed for interpretability and should be interpreted as a model-dependent descriptive explanation rather than evidence of expression direction, causal importance, or biological mechanism; no protein-, cell-type-, or functional-level validation was performed.

These limitations help frame the appropriate scope of the conclusions. They do not negate the methodological contribution of the study but define the level at which its findings should be interpreted. The present findings should be viewed as proof-of-concept evidence that targeted NanoString immune-response profiles carried a strong within-dataset measurement-level classification signal in this small longitudinal dataset. The study also suggests that a limited subset of genes may carry much of that discriminatory information and that *AICDA* and *ARHGDIB* were prioritized consistently across the two analytical frameworks within the analyzed dataset. However, before such a model-prioritized gene set could be considered for clinical translation, it would require validation in substantially larger, more diverse cohorts, preferably with participant-level partitioning, external validation, more heterogeneous control groups, and direct comparison with alternative host-response signatures developed from RNA-seq or qPCR-based panels. However, these potential translational implications should be regarded as hypothesis-generating only. The present study did not evaluate prospective diagnostic performance, clinical utility, or implementation in real-world clinical settings; therefore, the identified gene patterns and classification models should not be interpreted as providing clinically actionable diagnostic information.

Overall, the present study contributes to the COVID-19 host-response literature from both methodological and biological perspectives. As a methodological analysis, it illustrates the value of comparing FDFS and LEFS in a small-sample transcriptomic setting while distinguishing signature discovery from preprocessing-leakage-reduced internal performance evaluation. Biologically, it prioritizes *AICDA* and *ARHGDIB* as model-associated transcriptomic features within a targeted immune panel. Together, these findings provide exploratory evidence that a targeted whole-blood immune panel can contain a measurement-level classification signal and establish a hypothesis-generating basis for studies using independently sampled participants.

## 5. Conclusions

In this secondary analysis of a publicly available transcriptomic dataset, NanoString Human Immunology Panel-based whole-blood transcriptomic profiles showed strong within-dataset measurement-level discrimination between COVID-19 and healthy-control measurements. Across the FDFS and LEFS frameworks, LightGBM showed the most favorable overall performance, and SHAP analysis consistently identified AICDA and *ARHGDIB* as the dominant contributors to model output. These findings provide proof-of-concept evidence that a targeted immune-response transcriptomic panel can contain a compact classification signal in this setting. However, sample-level LOOCV with repeated longitudinal measurements does not constitute independent participant-level validation, and external validation in larger, clinically diverse cohorts comprising independently sampled participants is required before clinical interpretation or translation. Importantly, given the availability of only three independent COVID-19 participants, the identified gene signatures and the prioritization of *AICDA* and *ARHGDIB* should be considered hypothesis-generating rather than confirmatory biological findings. Their reproducibility and biological relevance require validation in substantially larger, independent participant-level cohorts. Moreover, because the comparator group consisted exclusively of healthy controls, the identified classification signal should not be considered COVID-19-specific until it is evaluated against other infectious, inflammatory, and respiratory disease groups. Finally, the potential clinical and translational implications of these findings should be regarded as hypothesis-generating only. The present study did not evaluate prospective diagnostic performance, clinical utility, or implementation in real-world clinical settings; therefore, the identified gene patterns and classification models should not be interpreted as providing clinically actionable diagnostic information.

## Figures and Tables

**Table 1:** Leave-one-out cross-validation classification metrics for FDFS framework.

Metrics	Random Forest	XGBoost	LightGBM
Accuracy	0.969	0.969	1.000
Balanced Accuracy	0.950	0.950	1.000
Sensitivity	1.000	1.000	1.000
Specificity	0.900	0.900	1.000
Precision	0.957	0.957	1.000
NPV	1.000	1.000	1.000
F1 Score	0.978	0.978	1.000
MCC	0.928	0.928	1.000
Cohen’s Kappa	0.925	0.925	1.000
ROC-AUC	1.000	0.900	1.000
PR-AUC	1.000	0.957	1.000
Brier Score	0.017	0.087	0.005
Log Loss	0.070	0.331	0.060

Note: Performance metrics are calculated across 32 measurement-level profiles rather than 32 independent biological observations. These profiles were obtained from 13 unique participants: 10 healthy controls contributing one measurement each and three COVID-19 participants contributing 22 longitudinal measurements. Accordingly, the reported sample-level metrics should be interpreted as internal measurement-level performance.

**Table 2:** SHAP importance ranking for the LightGBM model in FDFS framework (highest-contributing genes).

Gene	Mean|SHAP|	LightGBM Importance
*AICDA*	2.824	236
*ARHGDIB*	0.638	50
*CD19*	0.049	3
*BCL2*	0.033	4
*CCL22*	0.022	1
*CTNNB1*	0.019	1
*CD22*	0.014	2
*CCBP2*	0.012	2
*CD3E*	0.007	1

**Table 3:** Leave-one-out cross-validation classification metrics for LEFS framework.

Metrics	Random Forest	XGBoost	LightGBM
Accuracy	1.000	0.969	1.000
Balanced Accuracy	1.000	0.950	1.000
Sensitivity	1.000	1.000	1.000
Specificity	1.000	0.900	1.000
Precision	1.000	0.957	1.000
NPV	1.000	1.000	1.000
F1 Score	1.000	0.978	1.000
MCC	1.000	0.928	1.000
Cohen’s Kappa	1.000	0.925	1.000
ROC-AUC	1.000	0.900	1.000
PR-AUC	1.000	0.957	1.000
Brier Score	0.017	0.087	0.006
Log Loss	0.092	0.331	0.067

Note: Performance metrics are calculated across 32 measurement-level profiles rather than 32 independent biological observations. These profiles were obtained from 13 unique participants: 10 healthy controls contributing one measurement each and three COVID-19 participants contributing 22 longitudinal measurements. Accordingly, the reported sample-level metrics should be interpreted as internal measurement-level performance.

**Table 4:** SHAP importance ranking for the LightGBM model in LEFS framework (highest-contributing genes).

Gene	Mean|SHAP|	LightGBM Importance
*AICDA*	2.642	225
*ARHGDIB*	0.435	36
*AIRE*	0.186	14
*CTNNB1*	0.086	4
*C4BPA*	0.069	7
*CD19*	0.065	2
*ABL1*	0.054	6
*ITGB2*	0.045	2
*BCL2*	0.021	3
*CCR5*	0.019	1

**Table 5:** Participant-aware leave-one-COVID-participant-out sensitivity analysis.

Metrics	Random Forest	XGBoost	LightGBM
Accuracy	0.969	0.938	1.000
Sensitivity	1.000	0.955	1.000
Specificity	0.900	0.900	1.000
ROC-AUC	0.998	0.875	1.000
PR-AUC	0.998	0.904	1.000
Brier Score	0.049	0.103	0.019

Note: Although predictions were generated using participant-aware outer folds, the dataset comprises only 13 unique participants, including three COVID-19 participants. Therefore, these results should be interpreted as exploratory participant-aware sensitivity estimates rather than definitive participant-level validation.

**Table 6:** SHAP Importance and Descriptive Expression Patterns of the Top-Ranked Genes in the FDFS and LEFS Frameworks.

Gene	FDFS Mean|SHAP|	LEFS Mean|SHAP|	Healthy-Control Mean	COVID-19 Mean	COVID-19 − Control Difference, log2	Approximate Fold Change	Direction in COVID-19
*AICDA*	2.824	2.642	4.071	3.123	−0.948	0.518	Lower
*ARHGDIB*	0.638	0.435	12.677	12.026	−0.651	0.637	Lower
*CD19*	0.049	0.065	7.910	6.192	−1.718	0.304	Lower
*BCL2*	0.033	0.021	8.401	7.663	−0.738	0.599	Lower
*CTNNB1*	0.019	0.086	9.931	10.565	+0.634	1.551	Higher
*AIRE*	—	0.186	4.111	3.331	−0.780	0.582	Lower
*C4BPA*	—	0.069	4.972	3.138	−1.834	0.281	Lower
*ABL1*	—	0.054	6.356	6.824	+0.468	1.383	Higher
*ITGB2*	—	0.045	11.693	12.498	+0.805	1.747	Higher
*CCR5*	—	0.019	7.344	8.583	+1.239	2.361	Higher
*CCL22*	0.022	—	3.821	3.131	−0.690	0.620	Lower
*CD22*	0.014	—	8.537	6.780	−1.757	0.296	Lower
*CCBP2*	0.012	—	4.848	3.508	−1.340	0.395	Lower
*CD3E*	0.007	—	10.962	10.232	−0.73	0.603	Lower

Footnote: FDFS denotes full-dataset feature selection, whereas LEFS denotes leave-one-out cross-validation-embedded feature selection. Mean|SHAP| represents the mean absolute SHAP value and reflects the magnitude of each gene’s contribution to model predictions; it does not indicate the direction of expression change. Expression values are presented as normalized log2-transformed means. Approximate fold change was calculated as 2^(mean COVID-19 − mean healthy control)^. Because the 22 COVID-19 profiles consisted of repeated measurements from only three participants, these expression summaries are descriptive and should not be interpreted as participant-level differential-expression estimates.

## Data Availability

The data analyzed in this study are publicly available in the BioStudies/ArrayExpress repository under accession number E-MTAB-8871 at https://www.ebi.ac.uk/biostudies/arrayexpress/studies/E-MTAB-8871 (accessed on 12 July 2026). The processed normalized expression matrix, raw RCC files, and associated sample metadata are available from the same repository. No new datasets were generated in the present study.

## Supplementary Materials

The following supporting information can be downloaded at: https://www.mdpi.com/article/10.3390/v18091009/s1, Table S1: Gene-selection stability across the 32 LEFS folds. Selection frequency indicates the number and percentage of LEFS folds in which each gene was retained by Elastic Net feature selection.
