# Supplementary material for: Tree-Based Classification of COVID-19 Using NanoString Whole-Blood Immune-Response Profiles: Comparison of Full-Dataset and LOOCV-Embedded Feature Selection
Source: Viruses. 2026 Sep 13;18(9):1009. doi: 10.3390/v18091009 (PMC13611854; doi:10.3390/v18091009)
Supplement: Supplementary file 1 [file viruses-18-01009-s001.zip › viruses-4506889-supplementary.pdf]

Supplementary Table S1: Gene-selection stability across the 32 LEFS folds. Selection frequency indicates the number and percentage of LEFS folds in which each gene was retained by Elastic Net feature selection.

Gene	Selected folds (n/32)	Selection frequency	Selection (%)
AICDA	32	1,000	100,0
ARHGDIB	32	1,000	100,0
CCL15	32	1,000	100,0
CCL22	32	1,000	100,0
CCR5	32	1,000	100,0
CD19	32	1,000	100,0
CD1A	32	1,000	100,0
CD22	32	1,000	100,0
CD45RB	32	1,000	100,0
CD79A	32	1,000	100,0
CD97	32	1,000	100,0
CTNNB1	32	1,000	100,0
FYN	32	1,000	100,0
HLA-DQA1	32	1,000	100,0
HLA-DQB1	32	1,000	100,0
HRAS	32	1,000	100,0
IL1RL2	32	1,000	100,0
IL6R	32	1,000	100,0
JAK1	32	1,000	100,0
LILRB5	32	1,000	100,0
MAPKAPK2	32	1,000	100,0
MIF	32	1,000	100,0
MS4A1	32	1,000	100,0
PAX5	32	1,000	100,0
PTGER4	32	1,000	100,0
SLAMF6	32	1,000	100,0
TNFRSF13C	32	1,000	100,0
TNFSF12	32	1,000	100,0
TRAF4	32	1,000	100,0
TRAF6	32	1,000	100,0
ZAP70	32	1,000	100,0
ICAM2	31	0,969	96,9
IL1RAP	31	0,969	96,9
MBP	31	0,969	96,9

Gene	Selected folds (n/32)	Selection frequency	Selection (%)
CD3E	30	0,938	93,8
CD79B	30	0,938	93,8
BCL2	28	0,875	87,5
CCBP2	25	0,781	78,1
CCL26	25	0,781	78,1
TIRAP	16	0,500	50,0
HLA-DRA	15	0,469	46,9
IL2	15	0,469	46,9
IL29	15	0,469	46,9
PSMB10	14	0,438	43,8
EBI3	9	0,281	28,1
KLRAP1	8	0,250	25,0
C4BPA	5	0,156	15,6
NOS2	5	0,156	15,6
FCGRT	4	0,125	12,5
AIRE	3	0,094	9,4
IL12B	3	0,094	9,4
IL22RA2	3	0,094	9,4
IL4	3	0,094	9,4
CD40	2	0,063	6,3
IL19	2	0,063	6,3
PIGR	2	0,063	6,3
STAT6	2	0,063	6,3
CD209	1	0,031	3,1
CD55	1	0,031	3,1
RAG2	1	0,031	3,1
RELA	1	0,031	3,1
TFRC	1	0,031	3,1
VTN	1	0,031	3,1
XCL1	1	0,031	3,1
